# SDN-Defend: A Lightweight Online Attack Detection and Mitigation System for DDoS Attacks in SDN

**DOI:** 10.3390/s22218287

**Published:** 2022-10-28

**Authors:** Jin Wang, Liping Wang

**Affiliations:** College of Computer Science and Technology, Zhejiang University of Technology, Hangzhou 310023, China

**Keywords:** Software Defined Networking (SDN), distributed denial of service (DDoS), CNN-ELM, detection method, IP traceback, mitigation method

## Abstract

With the development of Software Defined Networking (SDN), its security is becoming increasingly important. Since SDN has the characteristics of centralized management and programmable, attackers can easily take advantage of the security vulnerabilities of SDN to carry out distributed denial of service (DDoS) attacks, which will cause the memory of controllers and switches to be occupied, network bandwidth and server resources to be exhausted, affecting the use of normal users. To solve this problem, this paper designs and implements an online attack detection and mitigation SDN defense system. The SDN defense system consists of two modules: anomaly detection module and mitigation module. The anomaly detection model uses a lightweight hybrid deep learning method—Convolutional Neural Network and Extreme Learning Machine (CNN-ELM) for anomaly detection of traffic. The mitigation model uses IP traceback to locate the attacker and effectively filters out abnormal traffic by sending flow rule commands from the controller. Finally, we evaluate the SDN defense system. The experimental results show that the SDN defense system can accurately identify and effectively mitigate DDoS attack flows in real-time.

## 1. Introduction

Distributed denial of service (DDoS) attack is a highly damaging distributed and large-scale coordinated network attack [1]. The attacker uses many puppet machines under its control to launch a denial of service (DoS) attack on the target simultaneously, which eventually causes the target system to run out of resources or even crash, making the target system unable to provide the required services to normal users. Since the first DDoS attack occurred in 1999, DDoS attack has become one of the most widespread and deadly cyber threats [2]. According to a survey report by Radware, DDoS attacks are currently the biggest network security threat faced by Internet-related organizations [3].

As a new network architecture, Software Defined Networking (SDN) [4,5] has the core idea of separating the data forwarding function of network equipment from the decision control function to realize the centralized control of the hardware. SDN provides sharing, flexibility, and fine-grained control over switches at a lower cost than traditional IP networks. SDN consists of a central controller with global visibility of the network state, and the communication between the controller and the switches is usually handled using the open and standard protocol OpenFlow [6], which allows the controller to update the flow rules in any switch directly once there is a demand. This simplified network architecture makes network control more flexible and enables SDN to be widely used in cloud data center network [7,8], wireless LANs [9,10], and cloud computing [11,12]. However, the centralized topology of SDN is vulnerable to DDoS attacks. DDoS attacks on traditional networks are generally initiated by botnets controlled by attackers. A large number of controlled endpoints consume the bandwidth and computational resources of the target resources by launching flooded application requests to the designated victims, forcing the target servers to stop normal application services [13]. In addition to this, researchers have also identified new DDoS attack techniques for SDN itself, such as packet_in flooding attacks against controllers [14], CrossPath attacks against southbound channels [15], and flow table overflow attacks against switches [16], etc. Both traditional DDoS attacks and new DDoS attacks targeting SDN architecture will seriously affect the performance of SDN. In recent years, many researchers have proposed many anomaly detection methods for DDoS attacks in SDNs [17,18,19]. These methods differ from traditional network anomaly detection methods by using the centralized deployment of Intrusion Detection Systems (IDS), which not only reduces the cost of adding additional detection devices but also improves the detection effectiveness.

At present, methods for anomaly detection of DDoS attacks in SDN are mainly divided into the following three categories: methods based on information statistics, methods based on machine learning, and methods based on deep learning [20]. Among the methods based on information statistics, the classical method is to use information entropy to calculate the changes in some characteristics of packets (such as the source/destination IP address of packets) to evaluate the abnormal situation of network traffic [21]. On the one hand, the detection accuracy of this method depends on the threshold value of entropy, but the selection of the threshold value depends on expert experience and the subjective judgment directly affects the detection accuracy. On the other hand, the method of information statistics is suitable for considering a small number of features; thus, it can easily lead to false detection. Subsequently, many researchers tried to use machine learning methods to detect DDoS attacks [22], such as support vector machines, decision trees, random forests, and other methods. Their performance is better than information statistical methods, but they have good results for processing low-dimensional features and small-sample data and are not suitable for high-dimensional and large-sample data detection. However, deep learning provides a good solution to the limitations of traditional machine learning. Deep learning methods (such as convolutional neural network, recurrent neural network, and graph neural network) can learn features and represent high-dimensional features into abstract data features, which can quickly and effectively process high-dimensional and large-sample data. At present, most of the anomaly detection methods based on deep learning use a single model, which cannot be well detected according to the characteristics of anomalous traffic and cannot guarantee real-time detection and detection accuracy. More importantly, existing DDoS attack defense methods only emphasize attack identification, with less research on mitigation strategies. On the one hand, some researchers migrate excessive anomalous traffic to other controllers for processing [23], which not only increases the workload of controllers in other domains but also increases the time delay, which is not beneficial for defending against DDoS attacks. On the other hand, other researchers put the normal traffic detected in the previous step into the self-built whitelist [24] and clear the abnormal traffic detected in the database that does not conform to the whitelist. However, none of the above mitigation methods consider the source of the attack, and eliminate the abnormal traffic from the source.

To solve the above problems, we propose a new defense mechanism for DDoS attacks based on SDN. The mechanism consists of a detection module and a mitigation module. The contributions of this paper are summarized as follows.

- The novelty of this paper is that it proposes an SDN defense system for online real-time detection and mitigation based on SDN for DDoS attacks. It combines the intrusion detection system of CNN-ELM with the IP traceback mechanism based on SDN architecture.
- The CNN-ELM intrusion detection method achieves higher detection accuracy compared to other methods, as shown in Figures 7–9.
- To effectively trace the source of attacks, a blacklist of abnormal traffic is established. Only abnormal packets detected by IDS are recorded, which saves memory space.
- The blacklist is designed to allow efficient IP traceback using the timestamp field of the packet/flow.
- To effectively mitigate DDoS attacks, anomalous flows are completely removed from the root cause by issuing flow table commands.

The rest of this article is structured as follows. Section 2 briefly introduces the research status; Section 3 presents the architecture and implementation details of our designed system. Section 4 describes the experimental steps and analyzes the experimental results. Section 5 provides conclusions and future work.

## 2. Related Work

With the development of Internet technology applications, the number of DDoS attacks is increasing greatly, one of the main reasons is the emergence of botnets. Attackers use malware to attack multiple hosts in the network and continuously send malicious traffic to the target hosts or servers, causing legitimate users to fail to access the network. As a new network architecture, SDN’s data plane and control plane are decoupled, which makes network control centralized and easy to manage, and provides a new idea for network security defense architecture. Therefore, SDN is widely used in cloud data center networks, wireless LANs, and cloud computing environments. In this section, we first introduce several DDoS attack detection methods in SDN, including statistical analysis, machine learning, and deep learning methods. Finally, the limitations of the above methods are analyzed, and our approach is proposed.

In recent years, many experts and scholars have proposed various detection methods for DDoS attacks in SDN, and the most common method is the statistical analysis-based anomaly detection method. The detection methods of statistical analysis take advantage of the property that normal traffic in the network follows certain statistical laws on certain characteristics [25,26], which can effectively distinguish all the traffic that does not conform to the law and treat this traffic as attack traffic. Commonly used statistical analysis detection methods include information entropy, principal component analysis, cardinality statistics, etc. Mousavi et al. [27] proposed an intrusion detection system that detects DDOS attacks by calculating entropy values in an SDN controller. Kalkan et al. [28] proposed a joint entropy-based DDoS attack detection method using SDN architecture features, which can mitigate not only known attack types but also unknown attack types. Salaria et al. [29] used an improved principal component analysis method to detect anomalous traffic in different classified regions. The experimental results showed that the detection accuracy reached 95.24%, which is 2.94% higher compared to the improved method. However, the statistical analysis method relies on a single fixed threshold, so it is easy to cause misjudgments of DDoS attacks. In addition, the threshold is different in different environments. Threshold adjustment needs rich experience. otherwise, it will directly affect the accuracy of detection. Based on the above two reasons, it is not reliable to use the statistical analysis method to judge the abnormal traffic on the actual network.

Machine learning is a typical traffic detection method, which can be divided into unsupervised learning and supervised learning. The difference between the two is whether the data samples used for model training contain classification labels or not. Commonly used unsupervised learning methods mainly include self-organizing mapping (SOM) [30] and K-means clustering [31]. To accurately detect DDoS attacks, Liu et al. [32] proposed a detection model Growing Hierarchical Self-Organizing Maps (GHSOM) with good adaptability and scalability, which can effectively identify unknown types of DDoS attacks. Unsupervised learning algorithms do not require a large number of samples to be labeled when training data, thus reducing the high cost of manual labeling. Therefore, it is suitable for classifying, analyzing, and mining potential relationships between large amounts of unlabeled data. In the field of anomaly detection, supervised learning has been widely deployed. Commonly used supervised machine learning methods include k-nearest neighbor (KNN) [33], support vector machine (SVM) [34], BP neural network [35], and so on. Wang et al. [36] proposed a DDoS attack detection method based on the BP neural network, which used the average number of bytes, the percentage of symmetric flows, the rate of change of asymmetric flows, and the percentage of small packets to training the classifier. However, the traditional machine learning methods described above are only suitable for the processing of low-dimensional and small-sample data, but not for the processing of high-dimensional and large-sample data.

In recent years, deep learning has developed rapidly and has achieved outstanding performance in computer vision and natural language processing. Therefore, researchers have started to apply it to the field of anomaly detection. Lin et al. [37] combined Let-Net5 with the softmax function for network anomaly classification. The authors used eight cross-validation techniques and successfully obtained an accuracy of 99.65%. Zhang et al. [38] proposed a two-stage anomaly traffic detection method for DDoS attack detection in SDN. The first stage uses the information entropy method to make coarse-grained judgments on abnormal traffic, and the second stage uses the deep learning hybrid model stacked sparse autoencoder (SSAE)—Support Vector Machine (SVM) to make fine-grained judgments on abnormal traffic. Through experimental verification, the method can identify more than 98% of DDoS traffic and the computational complexity and training time are reduced. Li et al. [39] proposed a deep-learning-based DDoS attack detection method Deep Convolution Neural Network (DCNN)—Deep Stacked Autoencoder (DSAE). The input features of this detection method consist of flow table features of the SDN switch and self-constructed flow table statistical features. Since it is a lightweight detection method, it can be deployed directly on the controller. Through experimental verification, this method has higher detection accuracy and a lower false alarm rate compared with the traditional machine learning methods of SVM and Deep Neural Network (DNN) methods. Yuan et al. [40] proposed a recurrent neural network (RNN) based DDoS attack detection method, DeepDefense. The detection model consists of CNN, RNN, and fully connected layers. Compared to traditional machine learning methods, DeepDefense reduced the error rate from 7.517% to 2.103% in Data15 and 39.69% in Data14. The Convolutional neural network (CNN) is a special feed-forward neural network that combines convolution and pooling operations to extract effective feature vectors from input data and improve the accuracy of classification, demonstrating the powerful potential of deep learning in anomalous traffic detection. Mahmoud et al. [41] took advantage of CNN feature extraction and proposed a new regularized adaptive method, SD-Reg, to solve the CNN overfitting problem. Additionally, the improved CNN model combined with the RF model is applied to the anomalous traffic detection of SDN, and the method improves the detection capability of the Network Intrusion Detection System (NIDS) for unknown events.

In summary, researchers have successfully applied different approaches in the field of anomaly detection. A large number of achievements have been made in DDoS attack detection, but there are still some pressing issues in this area. First, the current detection accuracy is not high enough for many practical application scenarios. Most approaches only emphasize improving accuracy or detection efficiency without optimizing both aspects at the same time. Then, in the research of DDoS attack defense in SDN, most methods only emphasize detection methods without considering how to mitigate abnormal traffic after it is detected. Therefore, in the next step, we will further explore how to effectively improve and optimize detection algorithms and abnormal traffic mitigation methods.

## 3. DDoS Attack Detection and Mitigation in SDN

### 3.1. Defense System Architecture Design

In this section, we describe the proposed SDN defense mechanism. The centralized management and programmability of SDN provide many advantages and facilitate the operation of SDN. Therefore, in this paper, we propose an SDN-based anomaly detection model for DDoS attacks and an IP address-based traceback mitigation method.

**Figure 1:**
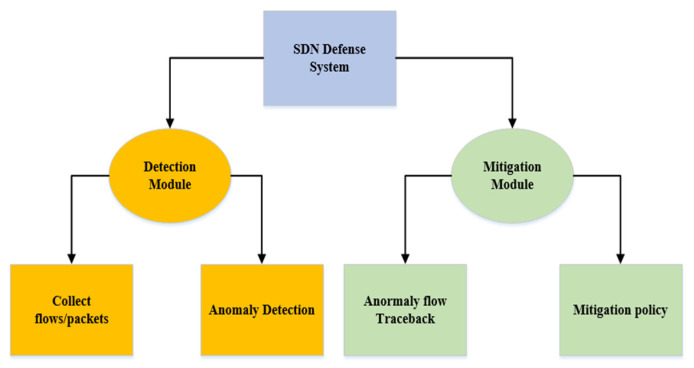
SDN security defense architecture module diagram.

Our proposed SDN defense system consists of two modules: a detection module and a mitigation module. They are deployed separately on the controller for logical communication, as shown in Figure 1. Each module is composed of two sub-modules. When the detection module detects DDoS attacks, the SDN defense system generates an alert, and when the controller receives the alert for anomaly detection, it automatically invokes the mitigation module to perform the corresponding operation. The specific implementation details of the detection and mitigation modules are presented in Section 3.4 and Section 3.5. Among them, the detection module can be divided into two sub-modules, the flow/packet collection module is used to collect packet_in packets and flow table rules in the switch; the anomaly detection module is to detect and process the collected data. Similarly, the mitigation module also has two sub-modules, the IP traceback module is to trace the abnormal traffic; the mitigation policy module is to contain the abnormal traffic from the attack source and mitigate the impact of the DDoS attack.

### 3.2. Defense System Architecture Design

**Figure 2:**
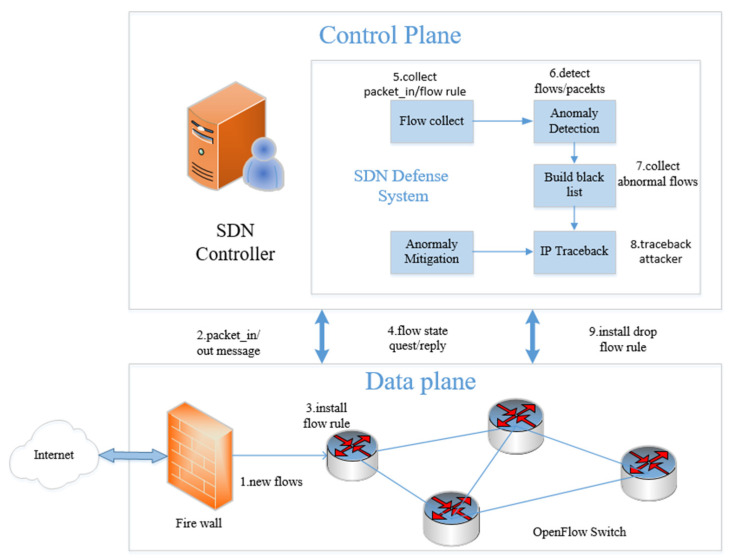
SDN security defense system flow chart.

The SDN defense system is deployed on the SDN controller, and its specific workflow is shown in Figure 2. When the packet_in event occurs, the SDN controller will extract the header feature fields of the packet_in packet with the ofp_packet_in command and send these fields to the controller’s exception detection module to determine if it is normal. If the packet is normal, it is indicated by 0; if it is abnormal, it is indicated by 1, and the packet information is sent to the blacklist. At the same time, every 1s, the SDN controller requests the flow table information to the switch through the ofp_flow_stats_reques command. Then, the controller sends the collected flow table information to the abnormal detection module, and the abnormal detection module represents the normal flow by 0 to carry out normal forwarding. Abnormal flows are represented by 1 and sent to the blacklist. Finally, the IP traceback method [42] improved in this paper is used to check the abnormal traffic information in the blacklist library, find out the control domain where the attacker resides, and send flow rules to the control domain through ofp_flow_mod message to block the attack source port, to reduce the impact of DDoS attacks in SDN. The workflow is shown in Algorithm 1.
**Algorithm 1:** SDN Defense System Algorithm**Input:** Traffic Sequence.**Output:** Send defense strategies.1: If   ofp_packet_in then2:       Packet_features = extract_features(ofp_packet_in)3:       Result=CNN-ELM(Packet_features)4:     If   Result ==0 then 5:          Packet_out message 6:     Else: Result ==1 then7:Log_blacklists(ingress_ip,src_ip,dst_ip,protocol,eth_type,src_mac,dst_mac,t_stamp,Controller_IP)8:     End if9: End if10:if   ofp_flow_states_reply then11:      Packet_features = extract_features(ofp_flow_stats_reply)12:         If   Result1 ==0 then13:               Packet forwarding14:         Else: Result1 ==1 then15:Log_blacklists(ingress_ip,src_ip,dst_ip,protocol,eth_type,src_mac,dst_mac,t_stamp,Controller_IP)16:         If   End if17: End if18: IP_traceback(Log_blacklists)19: Take_action(close the attack port)

### 3.3. Feature Construction

In machine learning methods for classification, the goodness of features has a large impact on the detection accuracy of the model, and good feature extraction requires complex arithmetic and empirical judgments. In contrast, in deep learning, the model can automatically extract features at different levels layer by layer, and combine these features at different levels to produce outputs.

Since the deep learning model is automatically extractable, some feature fields of the flow table in the switch are directly extracted as part of the first layer of input features to the model. The automatically obtained flow table features are shown in Table 1.

At the same time, to improve the detection accuracy of the model and ensure the reliability of the results, this paper manually constructs four statistical features to distinguish the abnormal traffic of DDoS attacks and takes them as another part of the input feature dataset of the model. The four manually constructed statistical features are shown in Table 2 and are described as follows.

1. The average growth rate of flow rules ($Avg_{flowSpeed}$). The network normal access, and flow rules in a certain period growth rate is more stable, whereas when suffering from a DDoS attack event, the number of flow rules will increase sharply, which may lead to flow table overflow and normal access being denied.
  (1) $${\mathrm{Avg}}_{flowSpeed}=\frac{\sum_{i=1}^{n}S_{i+1}-S_{i}}{n}$$
  where *t* denotes the sampling time interval; $S_{i}$ denotes the number of flow rules in the *t* time interval, and n denotes the number of sampling times.
2. Average survival time of flow rules ($Avg_{duration}$). To occupy target resources quickly and achieve the attack effect, flow rules of different IP addresses are rapidly added to the flow table in a short time. Compared with the normal user access time, the average access request time of the attacker is relatively short, and the lifetime of the flow rules will be reduced accordingly. Therefore, the average lifetime of flow rules becomes one of the important characteristics to distinguish DDoS from normal traffic.
  (2) $$Avg_{duration}=\frac{\sum_{i=1}^{m}duration_{i}}{m}$$
  where $duration_{i}$ denotes the duration of each flow rule, and m is the number of samples.
3. Average packet size ($Avg_{packetSize}$). DDoS attacks generally send fake packets to occupy the target victim’s resources or bandwidth resources. Usually, the average size of packets sent by attackers is much smaller compared with the size of packets sent by normal users in order to save attack cost; therefore, packet size is one of the important differences to distinguish DDoS attacks.
  (3) $$Avg_{packetSize}=\frac{\sum_{i=1}^{n}Psize_{i}}{n}$$
  where *Psize_i_* denotes the size of the packets collected during time interval *t*, and *n* is the number of samples.
4. Asymmetric flow Ratio ($Ratio_{asymmetricFlow}$). When a huge amount of traffic is flooded to the victim, the victim is usually unable to give feedback. Therefore, it results in an abnormal percentage of upstream and downstream traffic during DDoS. Both upstream and downstream packets are defined from the perspective of the switch. That is, packets from the switch to the endpoint are upstream traffic, and packets from the endpoint to the switch are downstream traffic. Therefore, when a DDoS attack occurs, the $Ratio_{asymmetricFlow}$ value of the port near the attack source is lower than 1.
  (4) $$Ratio_{asymmetricFlow}=Pbk_{i}/Pfd_{i}$$
  where *Pbk_i_* denotes the packets sent from the switch perspective, and *Pfd_i_* denotes the number of packets received from the switch perspective.

### 3.4. DDoS Attack Detection Model Based on CNN-ELM

CNN extracts object features mainly by multiple stacked convolutional and pooling layers, so CNN can classify features better. However, CNN networks use fully connected BP neural networks as the perceptron, and the network training uses gradient descent to find the network minimizing global error, which leads to a long time required for network training and poor network generalization, so the CNN perceptron is not a good classifier. ELM [43] is a single hidden layer feedforward neural network, in which the hidden layer weights and biases are randomly generated before training, and no adjustment is required during training, only the number of neurons in the hidden layer needs to be set to obtain the unique optimal solution, so it has the advantages of fast training speed, good generalization performance, and high classification accuracy. However, since ELM is a shallow neural network, its feature learning capability is limited, and high accuracy can only be obtained if the training data is good enough for the features. Because of the various excellent properties of convolutional neural network CNN and single-hidden layer feedforward neural network ELM, CNN and ELM are combined to build a CNN-ELM network to make full use of their respective advantages and overcome their disadvantages.

**Figure 3:**
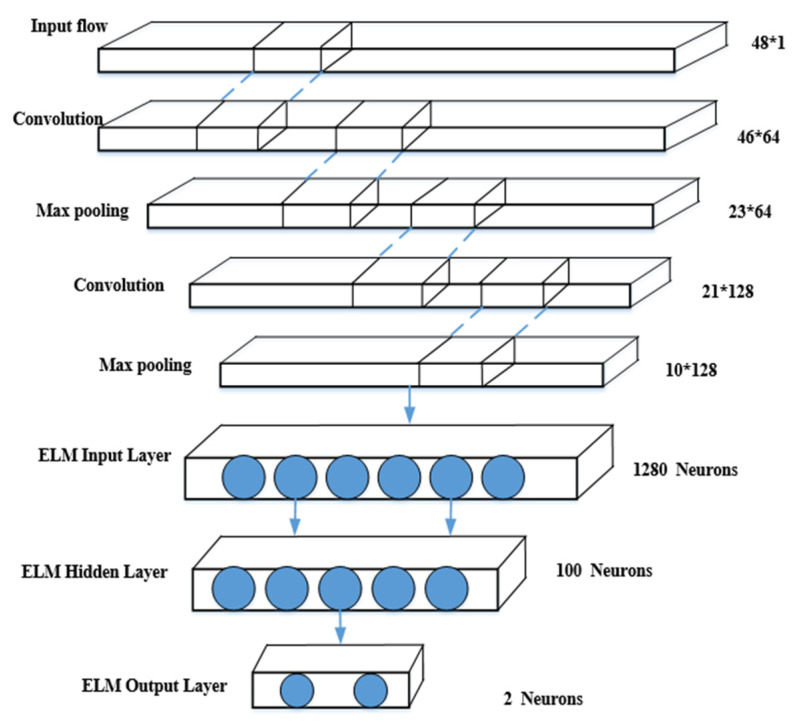
Deep learning hybrid model CNN-ELM.

In this section, we describe our hybrid model in detail. Figure 3 shows the architecture of our CNN-ELM. As can be seen from Figure 3, our network consists of two phases: feature extraction and classification. The stage of feature extraction includes convolutional layers and max pooling layers; the classification stage uses single hidden layer feedforward neural network ELM for classification. We also give the relevant parameters in detail, for example, the number of each filter, the size of each feature mapping, the kernel size of each filter, and the step size of each sliding window. For example, the first stage convolutional layer consists of 64 filters with a kernel size of 3 × 3 and a sliding window step size of 1, and the activation function is Relu. The second stage does a stride size of 2 and the kernel is a 2 × 2 maximum pooling, and the third stage convolutional layer consists of 128 filters with a kernel size of 3 × 3 and a sliding window with a step size of 1, and the activation function is Relu. The fourth stage does feature mapping which is a 2 × 2 maximum pooling. The fifth stage transforms the previous stage feature mapping into a one-dimensional vector and combines the ELM model to make it favorable for classifying DDoS attacks with normal flows, and we next describe in detail the design of the part of the hybrid model.

1. Convolution layer
  In the convolution layer, a series of convolution kernels convolve the input feature vectors to produce the corresponding feature maps, so there are several feature maps for each layer of convolution, but the same convolution kernel has the feature of sharing weights, which can effectively reduce the complexity of the model and reduce memory consumption. The convolution operation process as
  (5) $$z^{\left(l,m\right)}=f\left(w^{\left(l,m\right)}\times p^{\left(l-1\right)}+b^{\left(l,m\right)}\right)$$
  where, f represents the activation function, $w^{\left(l,m\right)}$ denotes the convolution kernel, $p^{\left(l-1\right)}$ represents the input of the previous layer, and $b^{\left(l,m\right)}$ is the bias term.
2. Pooling layer
  Also known as subsampling layer, it can compress the features of the convolutional layer, reduce the feature dimension while keeping the local invariance, reduce the network computation and effectively alleviate the phenomenon of network overfitting. Common pooling layers are classified as maximum pooling, average pooling, global average pooling, and global maximum pooling. In this paper, the maximum pooling function is selected, and its formula is expressed as
  (6) $$p^{l(i,j)}=\underset{(j-1)V+1\leq t\leq jV}{\max}\left\{a^{l\left(i,j\right)}\right\}$$
  where V denotes the pooling width, $a^{l(i,j)}$ denotes the activation function, and $p^{l(i,j)}$ denotes the output value.
3. Fully connected layer
  Its main role is feature classification, which can transform a two-dimensional feature vector into a one-dimensional feature vector by nonlinearly combining the features learned in the convolutional and pooling layers. Its mathematical description is
  (7) $$y=f\left(\sum_{i\in G_{i}}x^{\left(l-1,i\right)}w^{\left(l,i\right)}+b^{\left(l,j\right)}\right)$$
  There are many commonly used activation functions. Since the Relu function is simple to operate and fast to compute, it can effectively reduce the network time complexity and accelerate the convergence speed, and the Relu activation function is used in this paper.

1. ELM classification layer

**Figure 4:**
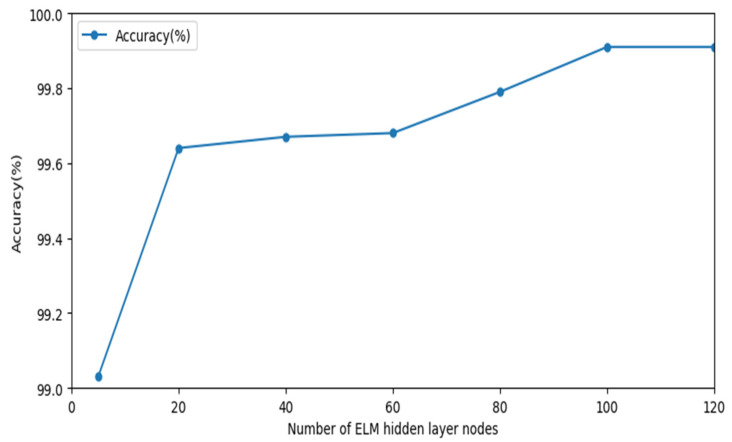
Anomaly detection accuracy for different number of nodes.

After the convolutional and pooling layers, the ELM method is used to classify the one-dimensional vectors transformed by feature mapping. The training mode of ELM is to generate input weights and hidden layer deviations randomly, and we only need to set the number of hidden layer neurons to calculate the output weights. Unlike the traditional training mode, it does not require iterative operations, which not only improves the training speed but also improves the generalization ability of the neural network. To determine the number of nodes in the hidden layer of the ELM network, this paper selects the Sigmoid activation function and the number of nodes in different hidden layers for experiments under the condition of CNN network structure. Experimental results are shown in Figure 4. When the number of hidden layer nodes is 100, relatively optimal anomaly detection accuracy can be obtained.

### 3.5. Mitigation Method Based on IP Traceback

**Figure 5:**
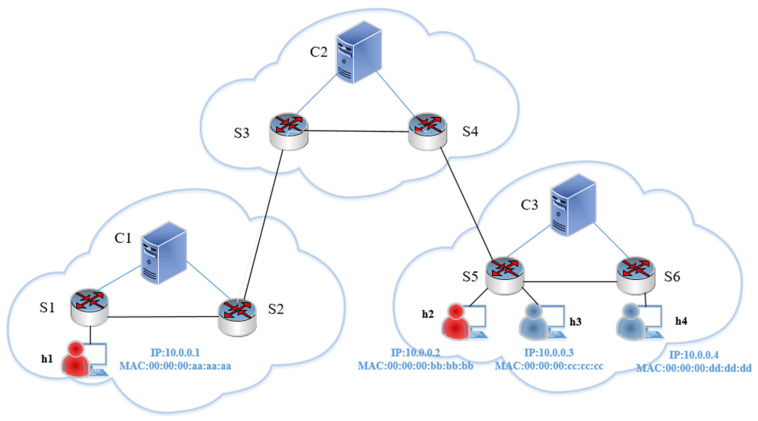
Network topological diagram.

**Figure 6:**
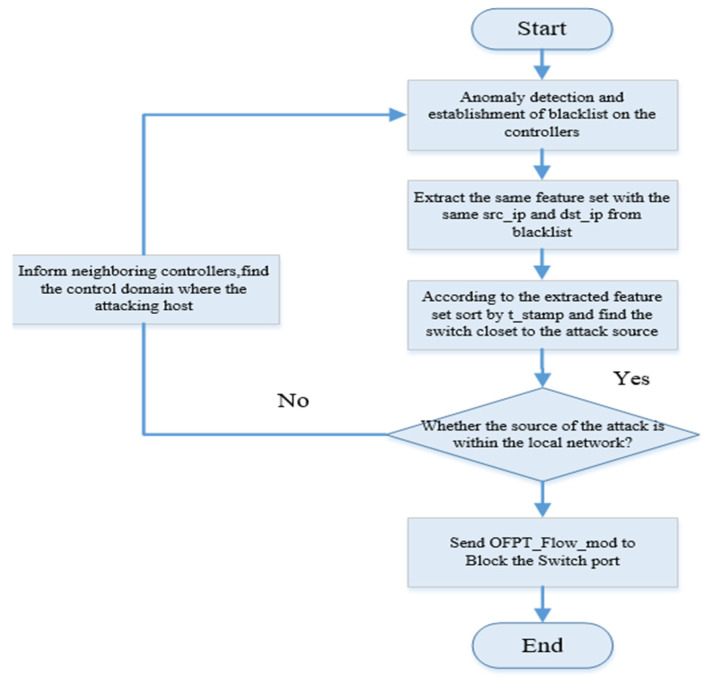
Flow chart of mitigation mechanism based on IP traceback.

To illustrate the IP traceback process, we implement simulation experiments using the network topology in Figure 5. The topology has three controllers, C1, C2, and C3, and four hosts, h1, h2, h3, and h4. Where h1 and h2 are the attacking hosts and h3 and h4 are the normal user hosts. h1 and h2 use the hping3 traffic generator to launch a DDoS attack on the target host h4. To effectively trace the source of the attack, the traceback attributes used in this paper are dpid, in_port, src_ip, dst_ip, protocol, eth_type, src_mac, dst_mac, origin, and t_stamp, respectively. Among them, the dpid and in_port attributes indicate the switch ID and the in_port number of the ofpt_packet_in message sent to the controller, respectively. Both attributes identify the ingress location of the packet into the network. src_ip, dst_ip, protocol, eth_type, src_mac, and dst_mac indicate the header fields of the packet. Origin is used to determine whether the source of the attack belongs to the current control domain. t_stamp indicates the timestamp when the ofpt_packet_in message arrives at the controller (the Source Mac and Destination Mac do not change from start to finish when packets are forwarded within the same control domain, which is crucial for our traceability determination). The above 10 attributes provide great help in tracing the path of the attack source. Suppose an attack occurs on a host in network 3, the SDN security defense system triggers the traceback module to store the abnormal flow in the blacklist in the controller according to the information of the above 10 attributes. The Source IP address and Destination IP address are used as the lookup objects and sorted according to the t_stamp attribute. If the attack packet comes from inside network 3, the traceback operation can show the complete path, and the algorithm flow is shown in Figure 6. The controller in network 3 will use the East–West interface to send messages to neighboring control domains to find the location of the packet source IP. Once the control domain where the attacking host is located is determined, this paper will sort the attack paths in order of the t_stamp attribute. Table 3 shows the attack traceback table for the source IP of 10.0.0.2 and destination IP of 10.0.0.4.

For cross-domain attacks, here we assume that the traceback source IP is 10.0.0.1 attack flow and the destination IP is 10.0.0.4 attack flow, and the traceback table of S5 in control domain C3 is shown in Table 4.

By using the above method, we can determine the control domain where the attacker is located, the first switch that forwards malicious traffic, and the switch’s ingress port number. Knowing the above information, the controller can block the ingress port of the first switch forwarding malicious traffic with the ofpt_flow_mod command to prevent malicious traffic from entering the network causing undesirable consequences.

## 4. Experimental Evaluation in the Detection and Mitigation of DDoS Attacks

### 4.1. Enviroment

To verify the detection model in this paper, we set up a simulation experiment environment. The Keras 2.2.4 [44] deep learning framework of TensorFlow-CPU 1.13 was used for simulation experiments. The operating system was Window 10, the Intel I5-6300HQ4 core processor was used, and the memory size was 8 G. It also uses an NVIDIA GTX960 graphics card to speed up the GPU. For the implementation of the attack traceback in this paper, we used Mininet 2.2.1 and OpenFlow 1.3, OpenvSwitch 2.7.0, and RYU 4.22. Mininet [45] is a network emulation orchestration system that runs a collection of switches, end hosts, routers, and network links. These network components are emulated on a single Linux kernel. The OpenFlow protocol is a network communication protocol that belongs to the data link layer and can control the forwarding plane of a network switch or router, thereby changing the network path taken by network packets. Open vSwitch [46] is a virtual switch capable of providing large-scale network automation using programmatic extensions. For the controller, we use RYU [47], a component-based SDN controller that provides well-defined APIs for software components, thus making it very easy for developers to create and test new network management and control applications.

### 4.2. Datasets

Intrusion detection system (IDS) performance relies heavily on the quality of the training dataset. However, the availability of benchmark datasets for intrusion detection is one of the main issues that will interrupt the development of anomaly detection systems. We can find a large number of datasets to evaluate different machine-learning techniques in different fields such as biomedicine, language translation, etc. However, privacy and security issues are the main reasons for the lack of network intrusion detection datasets. Network intrusion datasets are sensitive information, and once these datasets are made public, they will lose the credit of customers, so few network intrusion detection datasets are publicly available. In addition, most of the available datasets (such as the classic KDDCUP99, NSLKDD, etc.) are outdated and do not reflect the current network traffic trends. Additionally, some other datasets do not cover various known attack types and lack traffic diversity.

The CICIDS-2017 dataset [48] is one of the latest datasets available for intrusion detection. CICIDS-2017 dataset contains benign and latest common attacks such as brute force FTP, brute force SSH, DoS, Hearbleed, web attacks, infiltration, botnet, and DDoS attacks with data similar to real-world data (PCAPs) and the dataset has been processed into CSV format for easy use in intrusion detection. To effectively demonstrate the goodness of our proposed model, another dataset InSDN dataset (Elsayed et al.) [49] is also used in this paper to test the performance of the deep learning model proposed in this paper. This dataset covers recent common types of attacks such as DoS, DDoS, Brute Force Attacks, Malware, Probe, Exploitation, and Web attacks. In addition, the normal traffic in the InSDN dataset covers popular application services such as HTTPS, HTTP, DNS, Email, FTP, and SSH. The dataset simulates real attack scenarios, simulating SDN internal attacks with external attacks, using the CICFlowMeter open-source tool to extract more than 80 statistical features, containing 343,939 normal traffic and attack traffic, so the dataset is very similar to the attack data in the real network environment.

In this paper, to verify the performance of the proposed hybrid model CNN-ELM model for DDoS attack anomaly detection, the DDoS attack data collected on the fifth day in the dataset CICIDS-2017 is used to verify the goodness of the model. Additionally, DDoS attack data from the InSDN dataset are used to validate the hybrid CNN-ELM model. The dataset includes both DDoS attack traffic and legitimate traffic, and their distribution is shown in Table 5.

### 4.3. Feature Selection

The use of too many (useless) features in deep learning-based intrusion detection models may result in excessive computational costs and overfitting the training model to widely apply multiple attack detection. The use of fewer features cannot capture the attack characteristics, and the obtained intrusion detection models have low accuracy and a high false alarm rate, which can easily cause false positives. Therefore, the selection of features will directly affect the goodness of model training, and it is important to choose the appropriate features. In this paper, we minimize the cost and memory requirement of the hybrid intrusion detection model, and ensure high accuracy and running speed. In the experiments, this paper selects a subset of 12 features in the CICIDS-2017 and InSDN datasets concerning the features given in Section 3.3 to evaluate our model, as shown in Table 6. Additionally, compared with the 48 feature subsets proposed by Krishnan et al. [50] (50 feature subsets suggested in the original paper), the source IP and destination IP are removed because IP addresses can be forged, which will affect the training accuracy of the model, as shown in Table 7.

### 4.4. Data Pre-Processing

To build a real-time and effective intrusion detection system, we need to pre-process the data and reduce the complexity of the system operation before feeding them into the learning classifier. The specific work is as follows.

- Since the features have different ranges, it is necessary to rescale the data using the method of Equation (8). All data to between 0 and 1 are mapped.

(8) $$x_{scale}=\frac{x-x_{\min}}{x_{\max}-x_{\min}}$$

- For the experimental data, the data set is divided into 80% for training and 20% for testing using the train_test_split method of the Scikit-learn library.
- The labeled category uses the unique heat encoding technique to convert the label to a unique integer. The anomaly detection technology mentioned in this paper refers to binary classification technology. Therefore, in binary classification detection, the normal category is represented by binary 0, and the abnormal category is represented by binary 1.

### 4.5. Evaluation Metrics

To evaluate our proposed method, we used standard evaluation metrics to measure performance, namely confusion matrix, accuracy, precision, recall, and *F1* value. These metrics are calculated using four different measures in turn; true positive (*TP*), true negative (*TN*), false positive (*FP*), and false positive (*FN*), which are defined as follows.

*TP*: The number of samples whose actual type is DDoS attack, and the number of samples correctly judged by the detection model.

*TN***:** The number of samples whose actual type is normal and correctly judged by the detection model.

*FP*: The actual type of samples is normal, the number of samples misjudged by the detection model as DDoS attack type.

*FN*: The actual type of samples for DDoS attack, the number of samples misjudged as normal type by the detection model.

- Accuracy (*AC*): indicates the number of samples correctly judged by the detection model as a percentage of the total number of input samples.

(9) $$AC=\frac{TP+TN}{TP+TN+FP+FN}$$

- Precision (*P*): indicates the percentage of the number of samples judged by the detection model to be DDoS attack types that are DDoS attack types.

(10) $$P=\frac{TP}{TP+FP}$$

- Recall (*R*): indicates the number of samples correctly judged as DDoS attack types by the detection model as a percentage of the number of samples of all DDoS attack types.

(11) $$R=\frac{TP}{TP+FN}$$

- F_1_-score (*F*_1_): indicates the summed average of precision and recall, enabling a more accurate assessment of model performance.

(12) F1=21p+1R

- Confusion matrix: The confusion matrix gives a matrix as output and describes the complete performance of the model. It can be shown as a table with two dimensions, “actual” and “predicted”, and both dimensions have “true positive (*TP*)”, “true negative (*TN*)”, “false positive (*FP*)”, and “false negative (*FN*)”, as shown in Table 8.

### 4.6. Results and Analysis

#### 4.6.1. Analysis of Detection Mechanism Results

**Figure 7:**
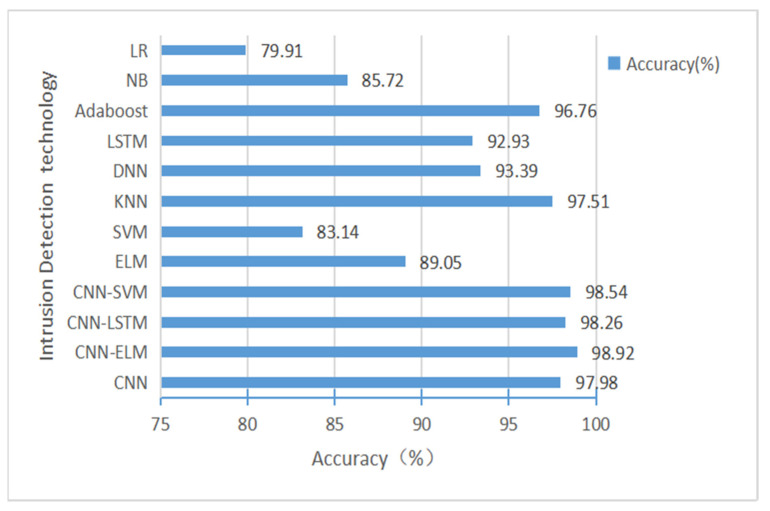
The accuracy comparison of CNN-ELM hybrid models based on the results of 12 subsets of features in the CICIDS-2017 dataset.

**Figure 8:**
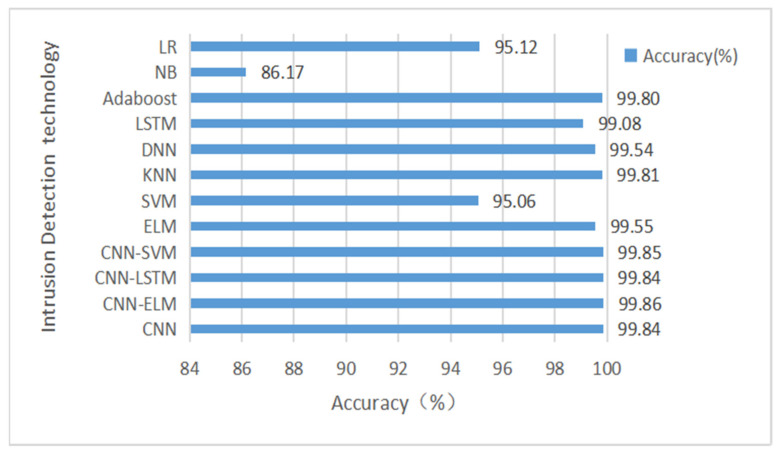
The accuracy comparison of CNN-ELM hybrid model based on the results of 48 subsets of features in the CIC-IDS 2017 dataset.

We used two datasets for testing: first, we trained and tested our model CNN-ELM using 12 feature subsets and 48 feature subsets from the CICIDS-2017 dataset, respectively, and the results obtained are compared with the results of other models as shown in Table 9 and Table 10. The accuracy comparison is shown in Figure 7 and Figure 8.

As can be seen from Table 9 and Table 10, the results for accuracy, recall, precision, and F1-score of the resulting CNN-ELM hybrid model outperformed the other machine learning models, both for the 12-feature subset and the 48-feature subset. In Table 10, although the accuracy improvement of the CNN-ELM model compared to the CNN, CNN-LSTM, and CNN-SVM models is not significant at 0.01–0.02%, the execution time of the CNN-ELM model is far better than the other models in terms of testing time.

From Figure 7 and Figure 8, we can see that the results of Accuracy of the resulting CNN-ELM hybrid model outperform other machine learning models for both the 12-feature subset and the 48-feature subset, indicating that the 12-feature subset selected in this paper can well reflect the characteristics of the attack traffic and is more concise and faster than the 48-feature subset in training the model.

**Figure 9:**
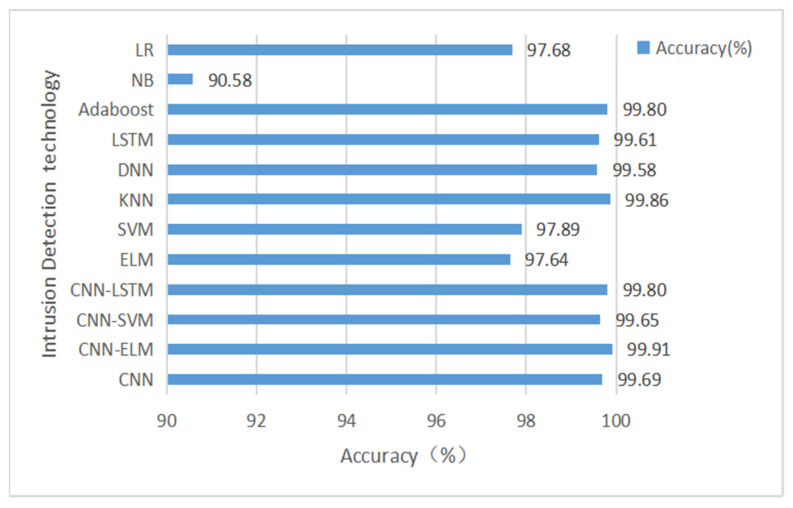
The accuracy comparison of CNN-ELM hybrid models based on the results of 12 subsets of features in the InSDN dataset.

To better reflect the wide applicability of the model proposed in this paper, we trained and tested the CNN-ELM model using the InSDN dataset with the 12 feature subsets proposed in this paper, and the experimental results compared with other machine learning models are shown in Table 11 and Figure 9.

From Table 11 and Figure 9, we can see that the CNN-ELM hybrid model outperforms the other machine learning models in terms of accuracy, recall, precision, F1-score and test time results for the InSDN dataset.

In summary, from the five aspects of detection accuracy, recall, accuracy, F1-score and testing time, CNN-ELM can quickly complete intrusion detection tasks when facing massive network data, and has high detection accuracy.

**Figure 10:**
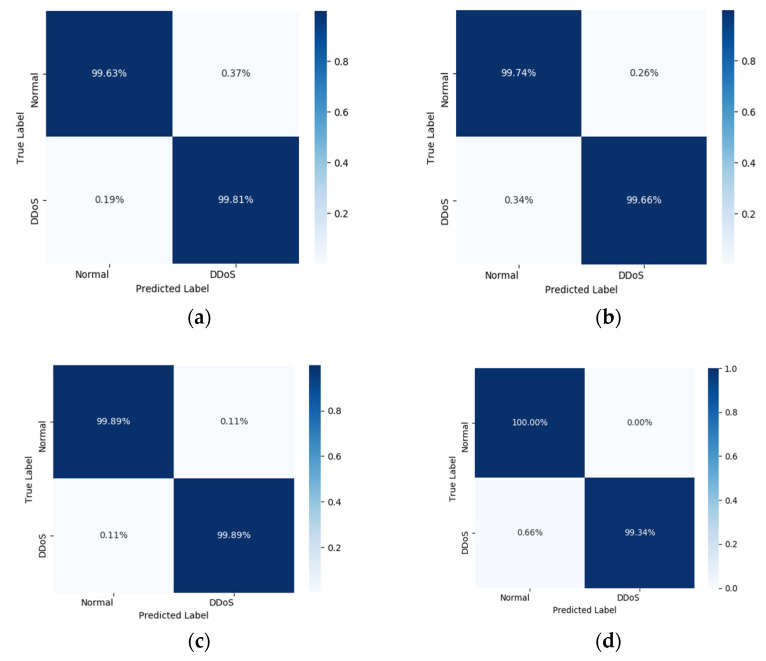
Comparison plot of the confusion matrix, (**a**) CNN 12 subset of features in InSDN; (**b**) CNN-LSTM 12 feature subset in InSDN; (**c**) CNN-ELM 12 subset of features in InSDN; (**d**) CNN-SVM 12 subset of features in InSDN.

To compare our models more intuitively, we compare the CNN, CNN-LSTM, and CNN-SVM models with the CNN-ELM hybrid model, respectively, and their confusion matrix plots are shown in Figure 10. Although the result of the CNN-SVM model is better than that of the CNN-ELM model proposed in this paper, the parameter setting of the CNN-SVM model is complex, it relies too much on expert experience and the training time of the model is long, so it is inferior to CNN-ELM model. On the whole, the CNN-ELM detection model proposed by us has a high detection rate and a low false positive rate, which is suitable for deploying in SDN for real-time DDoS attack detection.

#### 4.6.2. Analysis of Mitigation Mechanism Results

**Figure 11:**
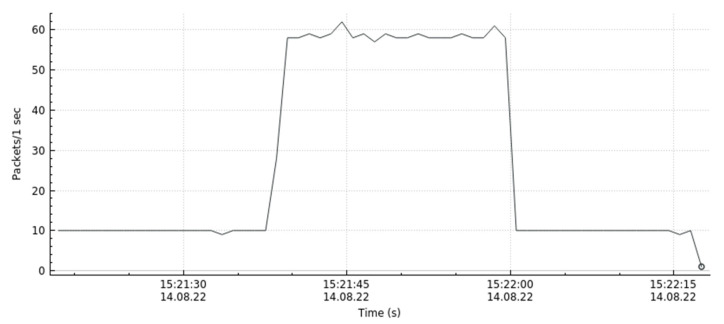
h3 Flow trend after defensive measures.

Based on the traceability method in Section 3.5 and topology Figure 5, we can initially determine that the attacker comes from switch S5 in control domain C3 and switch S1 in control domain C1 and then send flow rules commands to the attack source switch under this control domain to stop the malicious flow and observe the trend of traffic changes of the victim host h3. From Figure 11, we can see that at around 15:21:37, the attacker launches an attack on h3, and at around 15:22:00, the number of h3 packets drops and the traffic gradually returns to normal, indicating that the defense effect is successful.

## 5. Conclusions

This paper addresses the poor detection of DDoS attacks in SDN and proposes a deep learning hybrid model, CNN-ELM, which takes advantage of CNN to extract network traffic features and then uses the ELM algorithm for classification, which not only improves detection accuracy but also improves detection efficiency. To alleviate abnormal traffic, this paper uses the advantages of SDN global centralized control and management to trace the IP source of abnormal traffic, find the source of the attack, and inform the nearest controller to the victim to clear abnormal traffic, to curb DDoS attacks at the root. To verify the effectiveness of the SDN defense system, we conducted simulation experiments on the Mininet platform. The experimental results show that the proposed CNN-ELM model has good detection performance, and the accuracy obtained by hypothesis testing is 98.92% in the CICIDS-2017 dataset and 99.91% in the InSDN dataset. Meanwhile, the proposed SDN-based IP traceback method can effectively trace the source of attacks and mitigate DDoS attacks.

However, the anomalous traffic detection method proposed in this paper is based on a supervised learning method, which has the disadvantage that the cost of labeling the required data is very high. In future work, we hope to use unsupervised learning methods for anomaly detection of DDoS attacks in real network environments and explore the use of graph neural network [51] based method for attack traceback.

## Figures and Tables

**Table 1:** Flow table feature vectors.

Name	Describe
Src_ip	Source IP address
Dst_ip	Destination IP address
Src_mac	Source mac address
Dst_mac	Destination mac address
In_port	Input port
Src_port	Source port
Dst_port	Destination port
protocol	IP protocol
duration	Duration of Flow
ByteCount	bytes of flow
PacketCount	packets of flow

**Table 2:** Manually constructed statistical feature vectors.

Name	Describe
$Avg_{flowSpeed}$	average growth of flow rules
$Avg_{duration}$	Average duration of flow rules
$Avg_{\mathrm{packetSize}}$	Average size of packets
$Ratio_{\mathrm{asymmetricFlow}}$	Asymmetric flow Ratio

**Table 3:** IP traceback table for S5 in control domain 3.

Dpid	In_Port	Src_IP	Dst_IP	Protocol	Src_Mac	Dst_mac	Origin	Controller_IP	t_Stamp
5	1	10.0.0.2	10.0.0.4	6	00:00:00: bb:bb:bb	00:00:00: dd:dd:dd	Yes	192.168.1.1	15944782
5	1	10.0.0.2	10.0.0.4	6	00:00:00: bb:bb:bb	00:00:00: dd:dd:dd	Yes	192.168.1.1	15944872
5	1	10.0.0.2	10.0.0.4	6	00:00:00: bb:bb:bb	00:00:00: dd:dd:dd	Yes	192.168.1.1	15944992

**Table 4:** IP traceback table for S5 in control domain 3.

Dpid	In_Port	Src_IP	Dst_IP	Protocol	Src-Mac	Dst-Mac	Origin	Controller_IP	t_Stamp
5	1	10.0.0.1	10.0.0.4	6	00:00:00: aa:aa:aa	00:00:00: dd:dd:dd	No	192.168.1.1	15943782
5	1	10.0.0.1	10.0.0.4	6	00:00:00: aa:aa:aa	00:00:00: dd:dd:dd	No	192.168.1.1	15943872
5	1	10.0.0.1	10.0.0.4	6	00:00:00: aa:aa:aa	00:00:00: dd:dd:dd	No	192.168.1.1	15943992

**Table 5:** Flow distribution between CICIDS-2017 and InSDN dataset.

DataSet	Legitimate Flows	Attack Flows	Total
CICIDS-2017 (Friday-workingHours-DDoS)	97,718	128,027	225,745
InSDN (DDoS)	68,424	73,529	141,953

**Table 6:** Twelve subsets of features.

No.	Attribute Name	No.	Attribute Name
1	Fwd Pkt Len Mean	7	Protocol
2	Bwd Pkt Len Mean	8	Pkt Size Avg
3	Tot Fwd Pkts	9	Active Mean
4	Tot Bwd Pkts	10	Flow Duration
5	Src Port	11	Flow Byts/s
6	Dst Port	12	Flow Pkts/s

**Table 7:** Forty-eight subsets of features.

No.	Attribute Name	No.	Attribute Name
1	Flow Duration	25	Bwd IAT Total
2	Total Fwd Packets	26	Bwd IAT Mean
3	Total Backward Packets	27	Bwd IAT Std
4	Total Length of Fwd Packets	28	Bwd IAT Max
5	Total Length of Bwd Packets	29	Bwd IAT Min
6	Fwd Packet Length Max	30	Fwd Packets/s
7	Fwd Packet Length Min	31	Bwd Packets/s
8	Fwd Packet Length Mean	32	Packet Length Mean
9	Fwd Packet Length Std	33	Packet Length Std
10	Bwd Packet Length Max	34	Packet Length Variance
11	Bwd Packet Length Min	35	Packet Length Max
12	Bwd Packet Length Mean	36	Packet Length Min
13	Bwd Packet Length Std	37	Packet Size Avg
14	Flow Bytes/s	38	Active Mean
15	Flow Packets/s	39	Active Std
16	Flow IAT Mean	40	Active Max
17	Flow IAT Std	41	Active Min
18	Flow IAT Max	42	Idle Mean
19	Flow IAT Min	43	Idle Std
20	Fwd IAT Total	44	Idle Max
21	Fwd IAT Mean	45	Idle Min
22	Fwd IAT Std	46	Protocol
23	Fwd IAT Max	47	Fwd Header Length
24	Fwd IAT Min	48	Bwd Header Length

**Table 8:** Confusion matrix structure table.

Confusion Matrix		Actual Class	
		Positive	Negative
Predicted Class	Positive	*TP*	*FP*
	Negative	*FN*	*TN*

**Table 9:** Comparison results of CNN-ELM models of 12 feature subsets based on CICIDS-2017 dataset.

Method	Accuracy (%)	Recall (%)	Precision (%)	F1-Score (%)	Test Time (s)
CNN	97.98%	99.68%	96.52%	98.08%	4.26s
CNN-ELM	98.92%	99.67%	97.82%	98.74%	3.65s
CNN-LSTM	98.26%	99.29%	97.69%	98.48%	5.13s
CNN-SVM	98.54%	99.67%	96.92%	98.28%	34.56s
ELM	89.05%	97.39%	85.43%	91.02%	0.84s
SVM	83.14%	98.72%	77.70%	86.96%	26.13s
KNN	97.51%	98.53%	97.12%	97.82%	2.12s
DNN	93.39%	98.62%	90.61%	94.51%	0.78s
LSTM	92.93%	98.25%	90.21%	94.06%	2.29s
Adaboost	96.76%	98.88%	95.59%	97.21%	0.22s
NB	85.72%	99.14%	80.37%	88.77%	0.15s
LR	79.91%	98.16%	74.60%	84.77%	0.14s

**Table 10:** Comparison results of CNN_ELM models of 48 feature subsets based on CICIDS-2017 dataset.

Method	Accuracy (%)	Recall (%)	Precision (%)	F1-Score (%)	Test Time (s)
CNN	99.84%	99.84%	99.87%	99.86%	8.09s
CNN-ELM	99.86%	99.78%	99.89%	99.84%	4.70s
CNN-LSTM	99.84%	99.82%	99.89%	99.86%	6.37s
CNN-SVM	99.85%	99.75%	99.92%	99.84%	63.72s
ELM	99.55%	99.83%	99.37%	99.60%	0.81s
SVM	95.06%	99.50%	92.39%	95.81%	48.12s
KNN	99.81%	99.88%	99.78%	99.83%	6.32s
DNN	99.54%	99.83%	99.36%	99.59%	2.53s
LSTM	99.08%	99.80%	98.58%	99.19%	8.09s
Adaboost	99.80%	99.78%	99.87%	99.82%	0.31s
NB	86.17%	98.90%	80.97%	89.05%	0.19s
LR	95.12%	98.94%	92.93%	95.84%	0.23s

**Table 11:** Comparison results of CNN-ELM models with 12 feature subsets based on the InSDN dataset.

Method	Accuracy (%)	Recall (%)	Precision (%)	F1-Score (%)	Test Time (s)
CNN	99.69%	99.41%	100%	99.70%	2.17s
CNN-ELM	99.91%	99.89%	99.92%	99.91%	1.57s
CNN-SVM	99.65%	99.28%	100%	99.64%	16.76s
CNN-LSTM	99.80%	99.77%	99.85%	99.81%	1.34s
ELM	97.64%	95.61%	99.86%	97.69%	0.50s
SVM	97.89%	95.96%	100%	97.93%	0.51s
KNN	99.86%	99.88%	99.85%	99.87%	2.83s
DNN	99.58%	99.88%	99.32%	99.60%	0.47s
LSTM	99.61%	99.26%	100%	99.63%	2.69s
Adaboost	99.80%	99.65%	99.97%	99.81%	0.49s
NB	90.58%	99.60%	84.92%	91.68%	0.51s
LR	97.68%	95.54%	100%	97.72%	0.48s

## Data Availability

Not applicable.
